# Heterologous Production of the Structurally Complex Diterpenoid Forskolin in *Synechocystis* sp. PCC. 6803

**DOI:** 10.3390/bioengineering12070683

**Published:** 2025-06-23

**Authors:** Nadia Dodge, Lawrence Chuk Sutardja, Silas Mellor, Thiyagarajan Gnanasekaran, Lærke Marie Münter Lassen, Agnieszka Zygadlo Nielsen, Birger Lindberg Møller, Poul Erik Jensen

**Affiliations:** 1 Department of Food Science, University of Copenhagen, Rolighedsvej 26, 1958 Frederiksberg, Denmark; nadia.dodge@outlook.com; 2 Section for Molecular Plant Biology, Department of Plant and Environmental Sciences, University of Copenhagen, Thorvaldsensvej 40, 1871 Frederiksberg, Denmark; 3 Plant Biochemistry Laboratory, Department of Plant and Environmental Sciences, University of Copenhagen, Thorvaldsensvej 40, 1871 Frederiksberg, Denmark; silasmellor@plen.ku.dk (S.M.); blm@plen.ku.dk (B.L.M.); 4 Novo Nordisk Foundation Center for Basic Metabolic Research, University of Copenhagen, Blegdamsvej 3B, 2200 Copenhagen, Denmark; 5 VILLUM Center for Plant Plasticity, Thorvaldsensvej 40, 1871 Frederiksberg, Denmark

**Keywords:** metabolic engineering, light-driven production, cyanobacteria, diterpene synthases, cytochrome P450, acetyl transferase, labdane diterpenoid, *Plectranthus barbatus*, 13-R-Manoyl Oxide

## Abstract

Photosynthetic organisms such as cyanobacteria have the potential for the sustainable production of complex organic molecules due to their ability to use light as an energy source to fix CO_2_ and assimilate inorganic nutrients. Over the past decade, large efforts have been put into the metabolic engineering of cyanobacteria to produce various compounds such as alcohols, isoprenoids, biopolymers, and recombinant proteins. Forskolin is a structurally complex labdane-type diterpenoid with eight chiral carbon atoms and is naturally produced in the root cork of the plant *Plectranthus barbatus*. Forskolin is a potent cAMP activator indicated as a pharmaceutical for a variety of diseases. In the plant, forskolin biosynthesis from geranylgeranyl diphosphate involves six enzymes: two terpene synthases, three cytochrome P450s, and a single acetyltransferase. In this work, we express all six biosynthetic genes from *Plectranthus barbatus* in *Synechocystis* sp. PCC. 6803 and demonstrate heterologous production of this complex diterpenoid in a phototroph cyanobacterium. Forskolin titers reached 25.0 ± 4.4 µg/L and the forskolin was entirely secreted into the media. The forskolin-producing *Synechocystis* strain and empty vector control were cultivated in a photobioreactor for 8 days. Both strains showed similar chlorophyll *a* contents, and the forskolin-producing strain reached a slightly higher OD_730_ than the control. Forskolin began accumulating in the supernatant after 4 days and increased over time. These results indicate that forskolin production did not negatively impact cell growth.

## 1. Introduction

Plant natural products or specialized metabolites represent a rich and diverse array of compounds that humans have found useful for thousands of years. Terpenoids are a largest class of natural products with more than 170,000 compounds (http://terokit.qmclab.com/) used in different areas ranging from fragrances and flavorings to pesticides and pharmaceuticals [1]. In terms of production and market value, natural rubber, or polyisoprene, is one of the most important terpenoids, with 13.2 million tonnes produced and a USD 26 billion market value in 2017 [2]. The diterpenoid paclitaxel is an anti-cancer drug sold under the trade name Taxol^®^ and has a reported annual market value of over USD 1 billion [3]. These examples show terpenoids’ economic importance and provide a glimpse into the scale of demand. However, it is important to find more sustainable methods of production, especially for diterpenoids, which are typically produced in very low amounts in their host plants, are structurally complex, and are in ever-increasing global demand [4]. When target diterpenoids like forskolin, ginkgolides, and triptonide are exclusively produced in the roots of their host plants, digging out and harvesting the root material destroys the plants and has adverse consequences on soil erosion and demands extra areas of arable land when each harvest requires growth of a new generation of plants [5,6,7,8].

The heterologous production of plant-derived terpenoids in microorganisms is, therefore, an attractive alternative means of production that is independent of the use of arable land, does not result in soil erosion, and reduces the amounts of fossil fuel-based solvents and waste products associated with low-yield isolation from plant tissues harboring many other natural products. The biosynthesis of terpenoids in plants proceeds from orchestrated complex crosstalk between the two canonical C5-isoprene biosynthetic pathways: the mevalonate (MVA) pathway operating in the cytosol of the plant cell and the methylerythritol phosphate (MEP) pathway operating in the plastids [9,10]. Cyanobacteria, like chloroplasts, use the MEP pathway [11]. Both pathways converge with the production of isopentenyl diphosphate (IPP) and dimethylallyl diphosphate (DMADP). These C5-isoprene precursor molecules may be condensed to form C10 (monoterpenoids), C15 (sesquiterpenoids), C20 (diterpenoids), and a continuum of larger condensates.

The linear (C5)_n_ isoprenoids serve as substrates for terpene synthases, which catalyze the formation of cyclized core terpene structures of varying complexity. These are further decorated by oxygenations catalyzed by an assortment of cytochrome P450s (P450s) and by transferases. This results in complex regio- and stereospecific modifications, which complicate classical organic chemical synthesis [12]. P450s in eukaryotes are endoplasmic reticulum (ER) membrane-bound enzymes, dependent on electron donation from NADPH catalyzed by another membrane-bound protein, the cytochrome P450 oxidoreductase (POR) [13,14,15,16]. Cyanobacteria have attracted attention in recent years as heterologous production hosts as they merely require atmospheric CO_2_, inorganic elements, and light to grow and produce organic compounds [17,18,19]. Such potential for carbon-neutral or near-carbon-neutral production has accelerated the field of metabolic engineering in cyanobacteria [20,21,22]. Additionally, cyanobacteria occupy a wide range of ecological niches, allowing for the selection of species that grow in diverse non-arable locations [23]. As mentioned, P450s catalyze stereo- and regioselective oxygenations and require a dedicated reductase, as well as reducing equivalents like ferredoxin or NADPH, to be active. Both are produced by photosynthesis, rendering terpenoids prime targets for heterologous production in cyanobacteria. We and others have previously shown donation of electrons from photosystem I (PSI) to cytochrome P450s by endogenous soluble electron donors like ferredoxin and flavodoxin without the need for a dedicated reductase [24,25,26,27,28,29]. Of the two photosystems embedded in the thylakoid membranes, PSI is by far the most stable when exposed to photoinhibitory conditions protected by changes in cyclic electron transport around PSI [30]. The light-driven synthesis based on electron donation from PSI to the P450s is, thus, very robust and can support P450 turnover.

**Figure 1:**
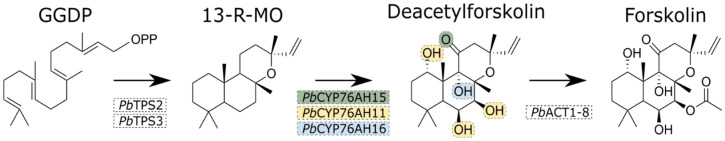
The forskolin biosynthetic pathway from the endogenous precursor GGDP. 13-R-MO: 13-R-Manoyl Oxide. Indicated are the two diterpene synthases (*Pb*TPS2 and -3), the three cytochrome P450s (*Pb*CYP76A11, -H15 and -H16) and the acetyl transferase (*Pb*ACT1-8) known to catalyze the final step in the biosynthesis of forskolin. The genes encoding all six enzymes were all obtained from *Plectranthus barbatus.* The reactions catalyzed by the individual cytochrome P450s, as identified by Pateraki et al. [6], are indicated in color.

In this study, we engineered *Synechocystis* sp. PCC. 6803 (hereafter *Synechocystis*) to produce forskolin. Forskolin is a bioactive structurally complex labdane-type diterpenoid containing eight chiral carbon atoms [31]. Forskolin can directly activate adenylate cyclase, leading to an increase in intracellular levels of the second messenger cyclic adenosine monophosphate (cAMP) [31]. Studies have implicated the use of forskolin in the treatment of heart failure and glaucoma and the induction of UV-less tanning, although approved clinical use has yet to be realized [32,33,34]. We show the production of forskolin by the expression of all six genes encoding the enzymes involved in the biosynthesis of forskolin in *Plectranthus barbatus* (previously known as *Coleus forskohlii*) (Figure 1) [5,6]. To the best of our knowledge, this is the first example of the production of a structurally complex diterpenoid in a cyanobacterial host.

## 2. Materials and Methods

### 2.1. Strains and Media

All cloning was performed using *E. coli* NEB 10-β carrying either pDF-trc [35] or pSL2387 [36] (a kind gift from Professor Himadri Pakrasi). The *E. coli* cells were grown in standard LB media supplemented with 50 µg/mL of spectinomycin or 50 µg/mL kanamycin. Conjugation was performed using the *E. coli* strain HB101 carrying the helper plasmid pRL443 grown in standard LB media supplemented with 100 µg/mL carbenicillin. Wild-type *Synechocystis* sp. PCC. 6803 and resulting strains were grown on solid BGH5 media or in liquid BGH11 media (BG5 or BG11 supplemented with 1.5 g/L or 4.75 g/L HEPES, respectively) [37].

### 2.2. Starter Cultures

Starter cultures of *Synechocystis* were grown from freezer stocks on plates at 30 °C at approximately 50 µmol photons m^−2^ s^−1^ irradiation using fluorescent lights. Liquid cultures were grown in culture tubes in a water bath at 30 °C with approximately 50 µmol photons m^−2^ s^−1^ fluorescent light with 3% CO_2_ (*v*/*v*) supplementation by bubbling.

### 2.3. Photobioreactor Cultures

Growth curves were performed in a Multi Cultivator MC 1000-OD (Photon System Instruments, Drásov, Czech Republic). Starter cultures (20 mL) were inoculated from plates and grown in liquid cultures, as described above. Initial 70 mL cultures were inoculated at 0.3 OD_730_ in BGH11 and supplemented with 100 µg/mL of kanamycin and spectinomycin. Cultures were flushed with 3% CO_2_ (*v*/*v*) and grown at 30 °C at a light intensity of 100 µmol photons m^−2^ s^−1^. OD at 730 nm was measured, and sample aliquots were collected at the start (day 1) and, subsequently, at days 2, 3, 4, 5, and 8. Cultures were induced on day 2 with 1 mM IPTG to ensure expression from the pDF-trc plasmid. Cells in the sample aliquots were harvested by centrifugation for further analysis.

### 2.4. Cloning and Transformation

All cloning was performed using DNA fragments amplified with Q5 polymerase (New England Biolabs, BioNordika Denmark A/S, Herlev, Denmark) and using the Hi-Fi DNA Assembly Kit (New England BioLabs). All genes were codon-optimized for expression in *Synechocystis* and obtained from GenScript. For 13-R-MO production, *PbTPS2* and *PbTPS3* from *Plectranthus barbatus* were used [5,38]. The TPS construct comprised *PbTPS2* and *PbTPS3* in an operon driven by the *Pcpc560* promoter [39] and integrated into the pSL2387 backbone containing the NSP1 [36] flanking homology regions, and a fully segregated strain expressing the two diterpene synthases was obtained, as reported in Sutardja et al. [40]. For experiments with the overexpression of the entire forskolin pathway, the *Plectranthus barbatus* genes encoding CYP76AH15, CYP76AH16, CYP76AH11, and ACT1-8, as described in [6,38], were expressed from an operon driven by the *Ptrc* promoter in pDF-trc plasmid backbone. The transformation of this plasmid into the TPS strain was performed by triparental mating, as described in [41].

### 2.5. Quantification of Forskolin

Samples were prepared by collecting 1.5 mL of the supernatant on days 1, 2, 3, 4, 5 and 8. Samples were concentrated to roughly 200 µL by vacuum centrifugation and extracted three times with 400 µL of ethyl acetate. The ethyl acetate was collected and evaporated to dryness by vacuum centrifugation, and the residue was resuspended in 80% MeOH spiked with 5 ppm andrographolide as an internal standard. The quantification of forskolin and deacetylforskolin was essentially performed as reported in Pateraki et al. [6]. In brief, LC-MS was performed using an Ultimate 3000 UHPLC Focused system ((Dionex Corporation, Sunnyvale, CA, USA) coupled to a Bruker Compact ESI-QTOF-MS ((Bruker Daltonik, Bremen, Germany) using a Kinetex XB-C18 column (100 × 2.1 mm i.d; 1.8 µm particle size, 100 Å pore size, Phenomenex Inc., Torrance, CA, USA). The column was maintained at 40 °C with a flow rate of 0.3 mL/min and a mobile phase consisting of (A) 0.05% (*v*/*v*) formic acid in water and (B) 0.05% formic acid in acetonitrile (*v*/*v*). The gradient used was as follows: 0–1 min isocratic 20% B; 1–23 min linear gradient to 100% B; 23–23.5 min linear gradient to 20% B; 23.5–27.5 min isocratic 20% B. The ESI source parameters were as follows: capillary voltage: 4500 V; dry gas flow: 8 L/min; dry gas temperature: 250 °C. The operation was set in MS/MS mode with the collision cell energy set to 7 eV and the RF set to 500 Vpp. Ions were monitored in positive mode at an *m*/*z* range of 50–1300 and spectra were collected at a rate of 2 Hz. The injection volume was 5 µL. Quantification was performed by calibration to authentic deacetylforskolin and forskolin standards (Sigma-Aldrich, Søborg, Denmark).

### 2.6. Chlorophyll Extraction and Quantification

Sample preparation for chlorophyll analysis was performed by taking 1.5 mL of culture on days 1, 2, 3, 4, and 8 of the photobioreactor run and harvesting the pellet. The pellet was resuspended in 1 mL of 95% MeOH flushed with nitrogen gas. The pigments were left for extraction in the dark in sealed tubes for 1 h with the headspace flushed with nitrogen gas. The resulting sample was filtered through a Durapore 0.22 µm PVDF centrifugal filter (Sigma-Aldrich, Søborg, Denmark). Pigment analysis was performed on a Shimadzu Prominence HPLC equipped with an SPD-M20A module using an Agilent Zorbax Extend-C18 column (150 mm × 4.6 mm i.d.; 2.5 µm particle size, Agilent Technologies, Glostrup, Denmark). The mobile phase consisted of (A) acetonitrile/methanol/0.1 M Tris-HCl pH 8.0 (84/2/14 *v*/*v*/*v*) and (B) methanol/ethyl acetate (68/32 *v*/*v*). A gradient between A and B was performed as follows: 0–20 min linear from 100 to 0% A; 20–26 min isocratic 0% A; 26–27 min linear 0–100% A; 27–38 min isocratic 100% A. The injection volume and flow rate were 40 µL and 1 mL/min, respectively. Peaks were detected and integrated at 445 nm for chlorophyll *a*. Chlorophyll *a* was identified by its absorption spectra, as well as its elution time.

## 3. Results and Discussion

The first committed step in forskolin biosynthesis is the conversion of GGDP to 13-R-Manoyl Oxide (13-R-MO) catalyzed by two diterpene synthases (TPSs) (Figure 1). Subsequently, five oxygenation events on the carbon skeleton catalyzed by three different P450s yield deacetylforskolin. Finally, an acetyltransferase (ACT) converts deacetylforskolin into forskolin [6].

**Figure 2:**
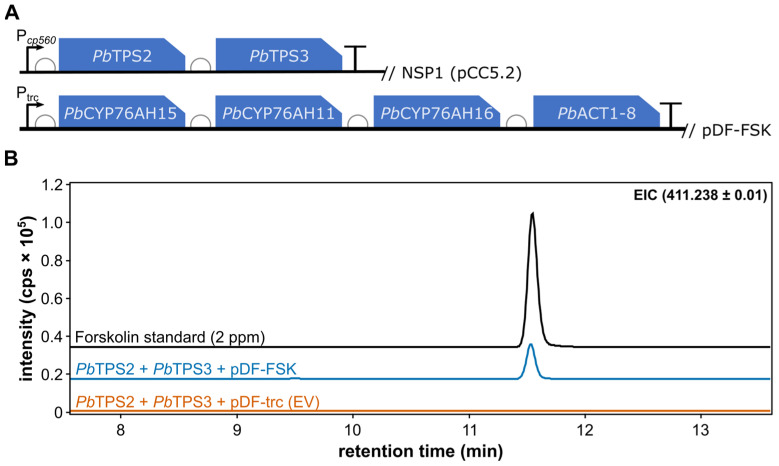
The heterologous production of forskolin in *Synechocystis*. (**A**) The *Synechocystis* strain FSK contains the following constructs: The TPS operon containing TPS2 and -3 is integrated into the NSP1-neutral site, and the operon containing the three P450s (CYP76AH15, CYP76AH11 and CYP76H16) and ACT1-8 resides on the replicative plasmid pDF-FSK (pDF-trc derivative). Promoters are indicated with an arrow (*Pcpc560* promoter for NSP1 and *Ptrc* promoter for pDF-FSK). The ribosomal binding sites (RBCs) are indicated with half circles. (**B**) LC-MS-based extracted ion chromatograms (EICs) showing the [M + H]^+^ adduct of forskolin in supernatants of cyanobacteria containing all pathway genes (*Pb*TPS2, *Pb*TPS3 and pDF-FSK) and the empty vector control (*Pb*TPS2, *Pb*TPS3 and pDF-trc (empty vector, EV)) are compared to the authentic forskolin standard.

A forskolin-producing *Synechocystis* strain was built by integration of the two terpene synthases driven by the P*cpc560* promoter [39] into the NSP1 neutral site [36,40], and by simultaneous expression of an operon containing the three P450s and the acetyl transferase driven by the P*trc* promoter on the replicative plasmid pDF-trc [35] (Figure 2A). Independent antibiotic-resistant colonies were selected and screened for forskolin production. 

Measuring the activity of individual enzymes in the pathway was not possible because the required intermediates are complex, challenging to synthesize, and difficult to isolate from natural sources due to their structural similarity to other intermediates, making purification impractical for enzymatic assays. Instead, we decided to analyse the production of the end-product forskolin, the compound previously documented to be synthesized by the six genes here introduced in *Synechocystis* sp. PCC 6803 [6]. Indeed, the strain containing the two diterpene synthases and the plasmid carrying the three cytochrome P450 genes and the acetyltransferase (*Pb*TPS2/3 and pDF-FSK) produce detectable forskolin after 8 days (Figure 2B). No forskolin could be detected in a corresponding strain containing the empty pDF-trc vector. These results demonstrate that the complete pathway is expressed and active in cyanobacteria.

**Figure 3:**
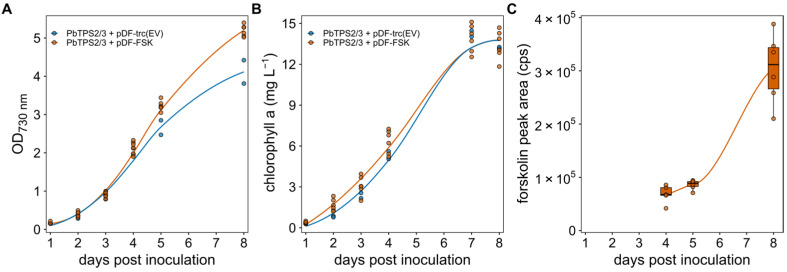
Growth curves, chlorophyll *a* contents, and forskolin accumulation in an 8-day photobioreactor growth experiment of *Synechocystis* strains containing the two diterpene synthases (PbTPS2, PbTPS3) and either the empty pDF-trc plasmid (pDF-trc (EV)) or the pDF-FSK plasmid expressing the three cytochrome P450 genes and the acetyltransferase. (**A**) OD_730nm_, (**B**) Chlorophyll *a* (mg/L), or (**C**) the peak area of the forskolin peak from the LC-MS ion chromatograms (cps, counts per second).

Transformants of the forskolin-producing strain (FSK), as well as an empty vector control, were grown in a photobioreactor, as outlined in the Section 2. Over 8 days of growth, the OD_730_ of the culture and chlorophyll *a* content of the cells were measured (Figure 3A,B). The FSK strain reached an OD_730_ of around 5 while the empty vector control reached an OD_730_ of around 4 after 8 days growth. The chlorophyll *a* content was quite similar in the two strains. The difference in the observed OD_730_ and chlorophyll contents can be explained by the fact that the forskolin-producing cells were slightly bigger). Forskolin accumulation was also measured on days 4, 5, and 8, and it was found to accumulate over time, becoming detectable in supernatant only after 4 days of cultivation. Overall, this suggests that the production of forskolin had no negative effect of the growth of the *Synechocystis* cells.

The expression of all six enzymes in the forskolin biosynthetic pathway in *Synechocytis* resulted in forskolin titers of 25 ± 4.4 µg/L in bioreactor cultures on day 8 with no forskolin detected in the control strain. Both the cell-free supernatant and the cell pellet were extracted to localize where the product accumulated. Forskolin was exclusively found in the cell-free supernatant, suggesting that all of it was secreted from the cells. Acetylforskolin, forskolin decorated with an additional acetyl group, was also identified in the supernatant but could not be quantified due to a lack of a genuine standard. In *Synechocystis* strains producing forskolin, the starting precursor 13-R-MO and the deacetylforskolin intermediate (Figure 1) could not be detected using GC-MS or LC-MS analysis, respectively, of the extracts. This could suggest that the production of forskolin in *Synechocystis* is limited by precursor supply, and future attempts to increase productivity should focus on increasing the supply of GGDP.

The fact that the forskolin is secreted is interesting. We have previously shown that the produced water-soluble compounds like dhurrin, *p*-hydroxyphenylacetaldehyde oxime, and aromatic amino acids in *Synechocystis* are secreted to the medium [26,42,43]. The ability of *Synechocystis* and possibly other cyanobacteria to secrete most of a desired produced compound facilitates the recovery of the produced compounds.

A yeast (*Saccharomyces cerevisiae)* strain expressing the entire forskolin biosynthetic pathway was able to produce up to 40 mg of forskolin per liter of yeast culture [6]. Thus, the need for improvements is evident and a prerequisite before photosynthetic organisms reach high production titers and commercial potential. However, we reported the production of the forskolin precursor 13-R-Manoyl Oxide (the product of the two diterpene synthases), which after 7 d in a photobioreactor increased to 880 µg/L [40]. Using a similar growth system, we, in the current study, observed production of 25 µg/L of forskolin, as well as an unknown amount of acetylforskolin. The production of simple terpenes in cyanobacteria are well-studied in the literature. Squalene, a triterpene (30-carbons), was produced in *Synechocystis* and reached a titer of 5.1 mg/L of culture in combination with overexpressing MEP pathway genes [44]. Using a high-density cultivation system with *Synechocystis*, the production of bisabolene, a sesquiterpene (15-carbons), reached 179.4 mg/L, and its oxidized form, bisabolol, reached 96.3 mg/L [45]. Even though it can be difficult to compare across different experimental setups, it is clear that *Synechocystis* has the potential to produce larger amounts of terpenes than the amounts of 13-R-Manoyl Oxide obtained in Sutardja et al. [40] and the amount of forskolin obtained in the current study. The reason for this is likely a combination of precursor supply, complexity of the enzymatic steps involved, and the potential toxicity of the products or one or more of the intermediates. In this study, we did not introduce the cytochrome P450 oxidoreductase that normally serves the electron donor to the P450s in *Plectranthus barbatus*. *Synechocystis* does not have a cytochrome P450 oxidoreductase, and it is therefore assumed that photo-reduced ferredoxin donates electrons to the three introduced P450s in the forskolin pathway, as shown previously [24,25,26,27,28,29].

In conclusion, we show that expression, in *Synechocystis* PCC. 6803, of the six genes encoding the enzymes involved in forskolin biosynthesis results in 25 µg/L of forskolin without the optimization of the photobioreactor conditions used. This represents the first engineered production of a structurally complex diterpenoid in a cyanobacterium. Precursor supply is hypothesized to be limited. Overall, we show the large potential for forskolin production and highlight steps needed to overcome the bottlenecks discovered in this study. Future work should be directed towards improving the supply of the diterpene precursor geranylgeranyl diphosphate, as well as optimizing the expression and needed stoichiometry of the three cytochrome P450s and the other enzymes in the pathway.

## Data Availability

The data that support the findings of this study are available from the corresponding author upon reasonable request.

## References

[1] Klaus O., Hilgers F., Nakielski AHasenklever D., Jaeger K.-E., Axmann I.M., Drepper T. (2022). Engineering phototrophic bacteria for the production of terpenoids. Curr. Opin. Biotechnol..

[2] Men X., Wang F., Chen G.Q., Zhang H.B., Xian M. (2019). Biosynthesis of natural rubber: Current state and perspectives. Int. J. Mol. Sci..

[3] El-Sayed A.S.A., Abdel-Ghany S.E., Ali G.S. (2017). Genome editing approaches: Manipulating of lovastatin and taxol synthesis of filamentous fungi by CRISPR/Cas9 system. Appl. Microbiol. Biotechnol..

[4] Sørensen M., Andersen-Ranberg JHankamer B., Møller B.L. (2022). Circular biomanufacture through harvesting solar energy and CO_2_. Trends Plant Sci..

[5] Pateraki I., Andersen-Ranberg J., Hamberger Britta Heskes A.M., Martens H.J., Zerbe P., Bach S.S., Møller B.L., Bohlmann J., Hamberger B. (2014). Manoyl oxide (13R), the biosynthetic precursor of forskolin, is synthesized in specialized root cork cells in *Coleus forskohlii*. Plant Physiol..

[6] Pateraki I., Andersen-Ranberg J., Jensen N.B., Wubshet S.G., Heskes A.M., Forman V., Hallström B., Hamberger Britta Motawia M.S., Olsen C.E., Staerk D. (2017). Total biosynthesis of the cyclic AMP booster forskolin from *Coleus forskohlii*. eLife.

[7] Forman V., Luo D., Lemcke R., Nelson D.R., Staerk D., Kampranis S., Møller B.L., Pateraki I. (2022). A gene cluster in *Ginkgo biloba* encoding for unique multifunctional cytochrome P450s orchestrates key steps in ginkgolide biosynthesis. Nat. Commun..

[8] Hansen N.L., Kjærulff LHeck Q., Forman V., Stærk D., Møller B.L., Andersen-Ranberg J. (2022). *Tripterygium wilfordii* cytochrome P450s catalyze the key methyl shift and epoxidations in biosynthesis of triptonide. Nat. Commun..

[9] Pattanaik B., Lindberg P. (2015). Terpenoids and their biosynthesis in cyanobacteria. Life.

[10] Mendoza-Poudereux I., Kutzner E., Huber C., Segura J., Eisenreich W., Arrillaga I. (2015). Metabolic cross-talk between pathways of terpenoid backbone biosynthesis in spike lavender. Plant Physiol. Biochem..

[11] Rohmer M. (2003). Mevalonate-independent methylerythritol phosphate pathway for isoprenoid biosynthesis. Elucidation and distribution. Pure Appl. Chem..

[12] Bathe U., Tissier A. (2019). Cytochrome P450 enzymes: A driving force of plant diterpene diversity. Phytochemistry.

[13] Jensen K., Jensen P.E., Møller B.L. (2012). Light-driven chemical synthesis. Trends Plant Sci..

[14] Jensen K., Møller B.L. (2010). Plant NADPH-cytochrome P450 0xidoreductases. Phytochemistry.

[15] Jensen K., Jensen P.E., Møller B.L. (2011). Light-driven cytochrome P450 hydroxylations. ACS Chem. Biol..

[16] Jensen S.B., Thodberg S., Parween S., Moses M.E., Hansen C.C., Thomsen J., Sletfjerding M.B., Knudsen C., Giudice R.D., Lund P.M. (2021). Biased cytochrome P450-mediated metabolism via small-molecule ligands binding P450 oxidoreductase. Nat. Commun..

[17] Liu D., Liberton M., Hendry J.I., Aminian-Dehkordi J., Maranas C.D., Pakrasi H.B. (2020). Engineering biology approaches for food and nutrient production by cyanobacteria. Curr. Opin. Biotechnol..

[18] Melis A., Martinez D.A.H., Betterle N. (2023). Perspectives of cyanobacterial cell factories. Photosynth. Res..

[19] Satta AEsquirol L., Ebert B.E. (2023). Current Metabolic Engineering Strategies for Photosynthetic Bioproduction in Cyanobacteria. Microorganisms.

[20] Dietsch M., Behle A., Westhoff P., Axmann I.M. (2021). Metabolic engineering of *Synechocystis* sp. PCC 6803 for the photoproduction of the sesquiterpene valencene. Metab. Eng. Commun..

[21] Bolay P., Dodge N., Janssen K., Jensen P.E., Lindberg P. (2024). Tailoring regulatory components for metabolic engineering in cyanobacteria. Physiol. Plantarum..

[22] Kukil K., Lindberg P. (2024). Metabolic engineering of *Synechocystis* sp. PCC 6803 for the improved production of phenylpropanoids. Microb. Cell Factories.

[23] Chorus I., Bartram J., Chorus I., Bertram J. (1999). Toxic Cyanobacteria in Water. A Guide to Their Public Health Consequences, Monitoring, and Management.

[24] Mellor S.B., Vinde M.H., Nielsen A.Z., Hanke G.T., Abdiaziz K., Roessler M.M., Burow M., Motawia M.S., Møller B.L., Jensen P.E. (2019). Defining optimal electron transfer partners for light-driven cytochrome P450 reactions. Metab. Eng..

[25] Nielsen A.Z., Ziersen B., Jensen K., Lassen L.M., Olsen C.E., Møller B.L., Jensen P.E. (2013). Redirecting Photosynthetic Reducing Power toward Bioactive Natural Product Synthesis. ACS Synth. Biol..

[26] Wlodarczyk A., Gnanasekaran T., Nielsen A.Z., Zulu N.N., Mellor S.B., Luckner M., Thøfner J.F.B., Olsen C.E., Mottawie M.S., Burow M. (2016). Metabolic engineering of light-driven cytochrome P450 dependent pathways into *Synechocystis* sp. PCC 6803. Metab. Eng..

[27] Berepiki A., Hitchcock A., Moore C.M., Bibby T.S. (2016). Tapping the unused potential of photosynthesis with a heterologous electron sink. ACS Synth. Biol..

[28] Berepiki A., Gittins J.R., Moore C.M., Bibby T.S. (2018). Rational engineering of photosynthetic electron flux enhances light-powered cytochrome P450 activity. Synth. Biol..

[29] Santos-Merino M., Torrado A., Davis G.A., Röttig A., Bibby T.S., Kramer D.M., Ducat D.C. (2020). Improved photosynthetic capacity and photoprotection via heterologous metabolism engineering in cyanobacteria. Proc. Natl. Acad. Sci. USA.

[30] Teicher H.B., Møller B.L., Scheller H.V. (2000). Photoinhibition of Photosystem I in field-grown barley (*Hordeum vulgare* L.): Induction, recovery and acclimation. Photosynth. Res..

[31] Alasbahi R.H., Melzig M.F. (2012). Forskolin and derivatives as tools for studying the role of cAMP. Pharmazie.

[32] D’Orazio J.A., Nobuhisa T., Cui R., Arya M., Spry M., Wakamatsu K., Igras V., Kunisada T., Granter S.R., Nishimura E.K. (2006). Topical drug rescue strategy and skin protection based on the role of Mc1r in UV-induced tanning. Nature.

[33] Majeed M., Nagabhushanam K., Natarajan S., Vaidyanathan P., Karri S.K., Jose J.A. (2015). Efficacy and safety of 1% forskolin eye drops in open angle glaucoma—An open label study. Saudi J. Ophthalmol..

[34] Mulieri L.A., Leavitt B.J., Martin B.J., Haeberle J.R., Alpert N.R. (1993). Myocardial force-frequency defect in mitral regurgitation heart failure is reversed by forskolin. Circulation.

[35] Guerrero F., Carbonell V., Cossu M., Correddu D., Jones P.R. (2012). Ethylene Synthesis and Regulated Expression of Recombinant Protein in *Synechocystis* sp. PCC 6803. PLoS ONE.

[36] Ng A.H., Berla B.M., Pakrasi H.B. (2015). 2015. Fine-tuning of photoautotrophic protein production by combining promoters and neutral sites in the cyanobacterium *Synechocystis* sp. strain PCC 6803. Appl. Environ. Microbiol..

[37] Stanier R.Y., Kunisawa R., Mandel M., Cohen-Bazire G. (1971). Purification and properties of unicellular blue-green algae (order Chroococcales). Bacteriol. Rev..

[38] Forman V., Bjerg-Jensen N., Dyekjær J.D., Møller B.L., Pateraki I. (2018). Engineering of CYP76AH15 can improve activity and specificity towards forskolin biosynthesis in yeast. Microb. Cell Factories.

[39] Zhou J., Zhang H., Meng H., Zhu Y., Bao G., Zhang Y., Li Y., Ma Y. (2014). Discovery of a super-strong promoter enables efficient production of heterologous proteins in cyanobacteria. Sci. Rep..

[40] Sutradja L.C., Dodge N., Walby S.L., Butler N.J., Gnanasekaran T., Møller B.L., Jensen P.E. (2024). Modulation of the MEP Pathway for Overproduction of 13-R-manoyl Oxide in Cyanobacteria. Synth. Biol. Eng..

[41] Casella S., Huang F., Mason D., Zhao G.-Y., Johnson G.N., Mullineaux C.W., Liu L.-N. (2017). Dissecting the Native Architecture and Dynamics of Cyanobacterial Photosynthetic Machinery. Mol. Plant.

[42] Lassen L.M.M., Nielsen A.Z., Olsen C.E., Bialek W., Jensen K., Møller B.L., Jensen P.E. (2014). Anchoring a plant cytochrome P450 via PsaM to the thylakoids in *Synechococcus* sp. PCC 7002: Evidence for light-driven biosynthesis. PLoS ONE.

[43] Brey L.F., Włodarczyk A.J., Bang Thøfner J.F., Burow M., Crocoll C., Nielsen I., Zygadlo Nielsen A.J., Jensen P.E. (2020). Metabolic engineering of *Synechocystis* sp. *PCC* 6803 for the production of aromatic amino acids and derived phenylpropanoids. Metab. Eng..

[44] Pattanaik B., Englund ENolte N., Lindberg P. (2020). Introduction of a green algal squalene synthase enhances squalene accumulation in a strain of *Synechocystis* sp. PCC 6803. Metab. Eng. Commun..

[45] Dienst D., Wichmann J., Mantovani O., Rodrigues J.S., Lindberg P. (2020). High density cultivation for efficient sesquiterpenoid biosynthesis in *Synechocystis* sp. PCC 6803. Sci. Rep..

